# Estimation of renal function in adult outpatients with normal serum creatinine

**DOI:** 10.1186/s13104-019-4487-6

**Published:** 2019-07-29

**Authors:** Temesgen Fiseha, Tizita Mengesha, Rahel Girma, Edosa Kebede, Angesom Gebreweld

**Affiliations:** Department of Clinical Laboratory Science, College of Medicine and Health Sciences, Wollo University, Dessie, Ethiopia

**Keywords:** Serum creatinine, Renal function, Estimated glomerular filtration rate

## Abstract

**Objective:**

The aim of this study was to estimate the prevalence of renal insufficiency using estimated glomerular filtration rate (eGFR) among adult outpatients with normal SCr.

**Results:**

A total of 414 patients with normal SCr were included in the study. Mean GFR (ml/min/1.73 m^2^) was 116.8 ± 43.5 using the MDRD equation and 90.5 ± 33.1 by the C–G formula. According to the MDRD formula, mild renal insufficiency (i.e. eGFR 60–89.9 ml/min/1.73 m^2^) was found in 21.5% of the patients and moderate renal insufficiency (i.e. eGFR 30–59.9 ml/min/1.73 m^2^) was found in 7.7%. According to the Cockcroft–Gault (C–G) formula, mild renal insufficiency was found in 38.2% and moderate renal insufficiency in 16.9% of the patients with normal SCr. In multivariate analysis, older age, female sex, a family history of kidney disease or other chronic diseases and high systolic blood pressure were associated with prevalent renal insufficiency depending on the formula used to estimate GFR. This study demonstrates the substantial prevalence of impaired renal function among Ethiopian adult outpatients with normal SCr. Including calculated estimates of GFR in routine laboratory reporting may help to facilitate the identification and thus optimal management of patients with renal insufficiency.

## Introduction

Kidney disease, even when renal function is only mildly or moderately altered, is an established risk factor for all-cause and cardiovascular mortality in high-risk patients, as well as in the general population [1–4]. Impaired renal function is also associated with an increased risk of several complications, such as anemia and bone mineral metabolism disorders, and poor outcomes, including cardiovascular events and progression to renal failure; requiring renal replacement therapy [5–7]. Early detection of renal impairment is, therefore, crucial to facilitate the employment of measures that can prevent or delay disease progression and reduce the risk of adverse outcomes [8]. Glomerular filtration rate (GFR) is accepted as the best indicator of renal function in both health and disease. GFR can be measured directly by clearance studies of ideal exogenous markers, such as inulin. However, none of these procedures are practical or economical for routine use and serum levels of endogenous filtration markers have traditionally been used to estimate renal function [9].

Serum creatinine (SCr) is the most widely used endogenous filtration marker for assessing renal function in clinical practice. However, SCr is insensitive to detect early renal disease, and levels could remain within the normal range even when renal function is significantly impaired; making the recognition and thus the optimal management of renal impairment at earlier stages more difficult [10, 11]. Current guidelines therefore recommend the use of prediction equations, such as the Modification of Diet in Renal Disease (MDRD) equation [12] and the Cockcroft–Gault equation [13], to estimate GFR (eGFR) whenever SCr is measured to facilitate early recognition of renal disease [14, 15]. In agreement with these guidelines, several studies provided evidence that incorporating eGFR into screening for renal impairment would identify individuals earlier in the natural history of the disease and enable the timely initiation of treatment to slow progression of renal disease and improve patient outcomes [16–18].

Most outpatient laboratories in Ethiopia do not routinely report estimated GFR when SCr is measured, and renal function is usually estimated by inspection of SCr levels in the primary care settings. Despite this, the under-ascertainment of impaired renal function among the Ethiopian adult outpatients with normal SCr has not been reported. Such information will be useful in establishing the clinical relevance and need of providing automated eGFR reporting for adult outpatients in the country. The aim of this study was to determine the prevalence of renal insufficiency using estimated GFR (eGFR) among adult outpatients with normal SCr at a hospital in Northeast Ethiopia.

## Main text

### Methods

#### Study design, setting and population

This cross-sectional study was conducted at the out-patient department of Debre Berhan Referral Hospital in North Shoa zone of Amhara regional state, which is located 130 km north of the capital Addis Ababa, Ethiopia. Adult outpatients (aged 18 years or more) referred by physicians for SCr measurements during the period from January to April 2018 were included in the study. Patients were excluded if they were treated with dialysis, hospitalized, have acute illnesses (fever) and if their SCr levels were abnormal (men > 1.5 mg/dl and women > 1.3 mg/dl). After applying exclusion criteria, 422 consecutive patients with normal SCr were qualified for the study.

#### Sample size determination

The sample size was calculated using single proportion formula on the basis of the following assumptions: a 95% confidence level; 5% margin of error; expected renal impairment prevalence of 50% and by adding 10% non-response rate. Respondents who did not participate in the examination component (n = 8) were excluded from the analysis. The final sample therefore included 414 individuals.

#### Data collection

Participants were interviewed for collecting demographic and other risk factor variables. Weight, height and blood pressure were measured at the time of the clinical examination performed. Body mass index (BMI) was calculated as weight square (kg) to height (meters), and participants were grouped into normal (BMI < 25 kg/m^2^), overweight (BMI = 25–29.9 kg/m^2^) and obese (BMI ≥ 30 kg/m^2^). Blood pressure (BP) was measured in the right upper arm in the sitting posture, after a 5 min rest and three measurements were averaged. Hypertension was defined as systolic BP ≥ 140 mmHg and/or diastolic BP ≥ 90 mmHg and/or use of antihypertensive medication. A blood sample was collected for SCr measurement using a modified Jaffe method as mg/dl with calibration traceable to IDMS reference material NIST SRM 909B level 2.

#### Measurement of renal function

Renal function was estimated according to the 4-variable Modification of Diet in Renal Disease (MDRD) study equation as eGFR = 186 × SCr (mg/dl)^−1.154^ × age (years)^−0.203^ × 0.742 (if female) × 1.210 (if black) [12] and the Cockcroft–Gault (C–G) formula [13] normalized for the body surface area (BSA): (140-age) × Weight (kg) × 0.86 (if female) × 1.73/72 × SCr (mg/dl) × BSA (m^2^). Patients were categorized as having normal renal function when the eGFR was ≥ 90 ml/min/1.73 m^2^, and mild, moderate and severe renal insufficiency when the eGFR was 60–89.9, 30–59.9 and 15–29.9 ml/min/1.73 m^2^, respectively [14, 19]. Moderate renal insufficiency was further categorized into G3a (eGFR 45–59.9 ml/min/1.73 m^2^) and G3b (eGFR 30–44.9 ml/min/1.73 m^2^) [19].

#### Statistical analysis

Statistical analyses were carried out using SPSS version 20.0 software (SPSS Inc., Chicago, IL, USA). Data were expressed as mean ± standard deviation (SD) or percentage. Comparisons between groups were done by Chi square (x^2^) test or *t*-test as appropriate. Multivariate logistic regression was conducted and the corresponding adjusted odds ratios (AOR) and 95% confidence intervals (CI) were used to identify factors independently associated with renal insufficiency. *P *< 0.05 was used to indicate statistical significance.

### Results

A total of 414 patients who had SCr level within the normal range participated in this study. Mean age was 48.9 ± 17 years, and 216 (52.2%) were males. Of the total participants, 83 (20.0%) were diabetic, 89 (21.5%) were hypertensive, 32 (7.7%) had cardiovascular disease and 29 (7.0%) were HIV positive patients. Majority, 372 (89.9%) of the participants had no a family history of kidney disease (FH-KD). Mean BMI was 21.82 ± 2.56 kg/m^2^. Mean systolic and diastolic BP (mmHg) were 124 ± 12 and 79 ± 10, respectively. Mean SCr was 0.88 ± 0.26 mg/dl. The mean eGFR (ml/min/1.73 m^2^) of the participants were 116.8 ± 43.5 and 90.5 ± 33.1 according to the MDRD and C–G equations, respectively (Table 1).

**Table 1:** Demographic and clinical characteristics of study subjects with normal serum creatinine (n = 414)

Characteristics	
Age (year), mean ± SD	48.9 ± 17
Age group, n (%)	
18–30	84 (20.3)
31–40	71 (17.1)
41–50	69 (16.7)
51–60	79 (19.1)
> 60	111 (26.8)
Sex, n (%)	
Male	216 (52.2)
Female	198 (47.8)
Education, n (%)	
< High school	292 (70.5)
≥ High school	122 (29.5)
Family history, n (%)	
Kidney disease	42 (10.1)
Hypertension, diabetes or CVD	90 (21.7)
Medical history, n (%)	
Diabetes	83 (20.0)
Hypertension	89 (21.5)
Cardiovascular disease	32 (7.7)
HIV	29 (7.0)
Current smoker, n (%)	23 (5.6)
Body mass index (Kg/m^2^), mean ± SD	21.82 ± 2.56
Systolic BP (mmHg), mean ± SD	124 ± 12
Diastolic BP (mmHg), mean ± SD	79 ± 10
Serum creatinine (mg/dl), mean ± SD	0.88 ± 0.26
eGFR_MDRD_ (ml/min/1.73 m^2^), mean ± SD)	116.8 ± 43.5
eGFR_C–G_ (ml/min/1.73 m^2^), mean ± SD	90.5 ± 33.1

BP blood pressure, *CVD* cardiovascular disease, *eGFR* estimated glomerular filtration rate

Estimation of renal function in patients with normal SCr using the MDRD and C–G equations are presented in Table 2. In patients with normal SCr, according to the MDRD equation mild renal insufficiency (eGFR 60–89.9 ml/min/1.73 m^2^) was found in 21.5% of the patients and moderate renal insufficiency (eGFR 30–59.9 ml/min/1.73 m^2^) was found 7.7%. According to the C–G formula, mild renal insufficiency was found in 38.2% of the patients and moderate renal insufficiency (eGFR 30–44.9 ml/min/1.73 m^2^) was found in 16.9% of the patients with normal SCr. Thirty-two (7.7%) and 38 (9.2%) of patients with normal SCr had mild to moderately impaired renal function (eGFR 45–59.9 ml/min/1.73 m^2^) according to the MDRD and C–G equations, respectively. In addition, 32 (7.7%) patients had moderate to severely impaired renal function (eGFR 30–44.9 ml/min/1.73 m^2^) despite normal SCr when renal function was estimated using the C–G formula (Table 2).

**Table 2:** Estimation of renal function in patients with normal serum creatinine using the simplified MDRD and Cockcroft–Gault formulas

GFR (ml/min/1.73 m^2^)	Description	MDRD, n (%)	Cockcroft–Gault, n (%)
≥ 90	Normal or high GFR	293 (70.8)	186 (44.9)
60–89.9	Mildly ↓ GFR	89 (21.5)	158 (38.2)
30–59.9	Moderate ↓ GFR	32 (7.7)	70 (16.9)
45–59.9	Mild to moderate ↓ GFR	32 (7.7)	38 (9.2)
30–44.9	Moderate to severe ↓ GFR	–	32 (7.7)

*GFR* glomerular filtration rate

Characteristics of patients with and without clinically significant renal insufficiency (eGFR < 60 ml/min/1.73 m^2^) according to the MDRD equation are shown in Table 3. Patients with renal insufficiency were significantly older, females, had low educational status, family history of kidney disease or other chronic disease (diabetes, hypertension or CVD), medical history of hypertension, high systolic and diastolic BP, higher BMI and SCr when compared with patients with eGFR ≥ 60 ml/min/1.73 m^2^. Except for gender and BMI, the same pattern was found when C–G formula was used.

**Table 3:** Characteristics of patients with and without renal insufficiency (eGFR _MDRD_ < 60 ml/min/1.73 m^2^)

	eGFR < 60 ml/min/1.73 m^2^	eGFR ≥ 60 ml/min/1.73 m^2^
Age above 60 years^b^, %	68.8	23.3
Female sex^b^, %	90.6	44.2
Education: < High school^a^, %	87.5	69.1
Family history, %		
Kidney disease^b^	28.1	8.4
HTN, DM or CVD^b^	50.0	19.4
Medical history, n (%)		
Hypertension^a^	43.8	19.6
Diabetes mellitus	31.2	19.1
Cardiovascular disease	9.4	7.6
HIV	6.2	7.1
Current smoker	9.4	5.2
Antihypertensive drug intake^b^, %	40.6	13.6
Systolic BP (mm Hg)^b^	134.1 ± 11.60	123.2 ± 11.56
Diastolic BP (mm Hg)^b^	85.6 ± 8.51	78.7 ± 9.52
Hypertension^b^, n (%)	65.6	27.5
BMI (kg/m^2^)^a^	22.87 ± 3.0	21.73 ± 2.5
Serum creatinine (mg/dl)^b^	1.34 ± 0.07	0.84 ± 0.23
eGFR (ml/min/1.73 m^2^)^b^	52.34 ± 2.65	122.19 ± 40.91

*HTN* hypertension, *DM* diabetes mellitus, *CVD* cardiovascular disease, *BP* blood pressure, *BMI* body mass index, *eGFR* estimated glomerular filtration rate

^a^Differences or associations significant at *P *< 0.05

^b^Differences or associations significant at *P *< 0.001

In multivariate analysis, older age (AOR = 10.81, 95% CI 4.05–28.83, *P *< *0*.001), female sex (AOR = 32.00, 95% CI 7.99–128.13; *P *< *0*.001), a family history of other chronic diseases (AOR = 3.06, 95% CI 1.19–7.86, *P *= *0*.020), and high systolic BP (AOR = 1.07, 95% CI 1.03–1.12, *P *= *0*.002) were independently associated with increased risk of renal insufficiency according to the MDRD equation. However, only older age (AOR = 14.06, 95% CI 7.39–26.77; *P *< *0*.001) and a FH-KD (AOR = 2.80, 95% CI 1.21–6.48, *P *= *0*.017) were independently associated with prevalent renal insufficiency when using C–G formula.

### Discussion

In this study, we found a high prevalence of abnormal renal function up to 55% on the basis of eGFR in adult out-patient with normal SCr. Clinically significant renal insufficiency (as defined by eGFR < 60 ml/min/1.73 m^2^) was found in 7.7–16.9% of the study participants depending on the formula used to estimate GFR. In previous studies, a considerable number of out-patients ranging from 5.3 to 19.3% have shown to have significantly impaired renal function (eGFR < 60 ml/min/1.73 m^2^) with normal SCr [20–23]. Another study also found that 13.9% of out-patients with normal range SCr levels had substantially abnor-mal calculated GFR, with C–G values < 50 ml/min [10]. These findings suggest that if SCr is used instead of eGFR as a measure of renal function, there is a likely chance of missing a significant number of patients with renal insufficiency.

This study shows that a large proportion of females and older persons with impaired renal function are not diagnosed if clinicians rely only on normal SCr levels as evidence of normal renal function. This is supported by the results of related studies and by the fact that SCr production is dependent on lean body mass and therefore may not be an accurate reflection of GFR, especially in older subjects and females because they have a reduced muscle mass [10, 16, 23–27]. The above studies have also demonstrated that inclusion of eGFR calculated by using equations which attempt to correct for factors affecting the muscle mass, such as age, sex and body size may facilitates the early identification and intervention of these subgroup of patients with renal impairment.

The finding of significantly prevalent renal insufficiency in patients with a FH-KD, a family history of other chronic diseases (diabetes, hypertension or CVD) and high systolic BP, suggests that a substantial proportion of cardiovascular at risk patients whose SCr levels fall within the normal range would not have been identified as having abnormal renal function without use of the GFR equations to estimate renal function. This is consistent with previous findings which documented that renal function should be assessed by using eGFR than SCr alone to facilitate the identification of high-risk patients with renal insufficiency at a time sufficient to ensure proactive care to delay disease progression and improve outcomes [21, 28, 29]. This was also supported by the NKF K/DOQI guidelines, which recommend using a GFR estimating equations to derive GFR from SCr (eGFR) rather than relying on SCr alone in at-risk populations [30].

Estimates of GFR using prediction equations provide substantial improvements over the measurement of SCr alone in the clinical assessment of renal function [31]. Several creatinine-based GFR prediction equations were developed in the past for estimating renal function, but the most commonly used are the MDRD [12, 32] and the C–G equations [13]. The MDRD equation that was developed using data from patients with established renal insufficiency as measured by ^125^I-iothalamate clearance adjusted for BSA is the most widely used in clinical practice today. Since it relies on age, sex, race and SCr only, this equation is quick and easy to calculate on all patients using data routinely provided when requesting a SCr measurement. It has been generally shown to provide a more accurate estimates of GFR than measured creatinine clearance or the C–G equation [12, 33]. The C–G equation, which predicts creatinine clearance [13], is a simple and recommended means to assess renal function. Unlike the MDRD equation, it requires a measure of height and computation of BSA (making it a less convenient method for routine use). However, eGFR derived from the C–G equation is superior to SCr alone in the assessment of renal function [33].

### Conclusions

In conclusion, this study demonstrates a substantial prevalence of renal impairment among Ethiopian adult outpatients identified as having normal SCr levels. A large proportion of the elderly, women and cardiovascular at risk patients will not be recognized as having impaired renal function if clinicians rely on normal SCr as evidence of normal renal function. Including calculated estimates of GFR in routine laboratory reporting may help to facilitate the early identification and thus optimal management of patients with renal impairment.

## Limitations

The use of calculated GFR but not measured GFR, which is not the gold standard, to estimate renal function is the first limitation. Secondly, we used the MDRD study equation, the validation of which is lacking among Ethiopian adults. Third, the measurement of serum creatinine was not standardized; this might influence the performance of eGFR equations, particularly at higher values. This also limited us from using the popular Chronic Kidney Disease Epidemiology (CKD–EPI) equation, as recommended by the KDIGO guidelines. Finally, the estimation of abnormal GFR is based on a single measurement of SCr which might lead to over or under estimating patients with abnormal GFR.

## Data Availability

The date of this study can’t be shared publicly due to presence of sensitive (confidential) participants’ information and additional data than that used in this publication. But the data are available from the corresponding author on reasonable request.

## Abbreviations

AOR: adjusted odds ratio
BMI: body mass index
BP: blood pressure
C–G: Cockcroft–Gault
CI: confidence interval
eGFR: estimated glomerular filtration rate
FH-KD: family history of kidney disease
GFR: glomerular filtration rate
MDRD: modification of diet in renal disease
